# Correlation of the Commercial Anti-SARS-CoV-2 Receptor Binding Domain Antibody Test with the Chemiluminescent Reduction Neutralizing Test and Possible Detection of Antibodies to Emerging Variants

**DOI:** 10.1128/Spectrum.00560-21

**Published:** 2021-12-01

**Authors:** Yoshitomo Morinaga, Hideki Tani, Yasushi Terasaki, Satoshi Nomura, Hitoshi Kawasuji, Takahisa Shimada, Emiko Igarashi, Yumiko Saga, Yoshihiro Yoshida, Rei Yasukochi, Makito Kaneda, Yushi Murai, Akitoshi Ueno, Yuki Miyajima, Yasutaka Fukui, Kentaro Nagaoka, Chikako Ono, Yoshiharu Matsuura, Takashi Fujimura, Yoichi Ishida, Kazunori Oishi, Yoshihiro Yamamoto

**Affiliations:** a Department of Microbiology, Toyama University Graduate School of Medicine and Pharmaceutical Sciences, Toyama, Japan; b Department of Virology, Toyama Institute of Health, Toyama, Japan; c Toyama City Hospital, Toyama, Japan; d Department of Clinical Infectious Diseases, Toyama University Graduate School of Medicine and Pharmaceutical Sciences, Toyama, Japan; e Laboratory of Virus Control, Center for Infectious Disease Education and Research (CiDER), Osaka University, Osaka, Japan; f Laboratory of Virus Control, Research Institute for Microbial Diseases (RIMD), Osaka University, Osaka, Japan; g Toyama Institute of Health, Toyama, Japan; University of Cincinnati

**Keywords:** neutralizing antibodies, seroconversion, receptor-binding domain, convalescent, high throughput

## Abstract

Serological tests are beneficial for recognizing the immune response against severe acute respiratory syndrome coronavirus-2 (SARS-CoV-2). To identify protective immunity, optimization of the chemiluminescent reduction neutralizing test (CRNT) is critical. Whether commercial antibody tests have comparable accuracy is unknown. Serum samples were obtained from COVID-19 patients (*n* = 74), SARS-CoV-2 PCR-negative (*n* = 179), and suspected healthy individuals (*n* = 229) before SARS-CoV-2 variants had been detected locally. The convalescent phase was defined as the period after day 10 from disease onset or the episode of close contact. The CRNT using pseudotyped viruses displaying the wild-type (WT) spike protein and a commercial anti-receptor-binding domain (RBD) antibody test were assayed. Serology for the B.1.1.7 and B.1.351 variants was also assayed. Both tests concurred for symptomatic COVID-19 patients in the convalescent phase. They clearly differentiated between patients and suspected healthy individuals (sensitivity: 95.8% and 100%, respectively; specificity: 99.1% and 100%, respectively). Anti-RBD antibody test results correlated with neutralizing titers (*r* = 0.31, 95% confidence interval [CI] 0.22–0.38). Compared with the WT, lower CRNT values were observed for the variants. Of the samples with ≥100 U/mL by the anti-RBD antibody test, 77.8% and 88.9% showed ≥50% neutralization against the B.1.1.7 and the B.1.351 variants, respectively. Exceeding 100 U/mL in the anti-RBD antibody test was associated with neutralization of variants (*P* < 0.01). The CRNT and commercial anti-RBD antibody test effectively classified convalescent COVID-19 patients. Strong positive results with the anti-RBD antibody test can reflect neutralizing activity against emerging variants.

**IMPORTANCE** This study provides a diagnostic evidence of test validity, which can lead to vaccine efficacy and proof of recovery after COVID-19. It is not easy to know neutralization against SARS-CoV-2 in the clinical laboratory because of technical and biohazard issues. The correlation of the quantitative anti-receptor-binding domain antibody test, which is widely available, with neutralizing test indicates that we can know indirectly the state of acquisition of functional immunity against wild and variant-type viruses in the clinical laboratory.

## INTRODUCTION

Understanding the status of immunity to severe acute respiratory syndrome coronavirus 2 (SARS-CoV-2) will help us overcome clinical problems created by the coronavirus disease-2019 (COVID-19) pandemic. Serological tests can provide information on immune status after viral exposure and vaccination. While the virus neutralizing test is a method for directly determining immune function, it is not suitable as a routine test in clinical laboratories due to its complexity and the risks associated with using live viruses. Therefore, commercially available antibody tests may help indirectly identify protective immunity.

We previously established the chemiluminescence reduction neutralization test (CRNT) for the evaluation of immunity to SARS-CoV-2, using pseudotyped virus (1). The CRNT assesses inhibition by serum samples on viral attachment and entry into target cells. As observed in our previous studies (1, 2), reduction of infectivity by sera from symptomatic COVID-19 patients gradually increased during the follow-up period, suggesting that the CRNT reflects the status of immunity acquisition.

Meanwhile, commercial antibody tests that do not assay for functional antibodies are becoming available in clinical microbiology. Some tests detect antibodies specific to the receptor-binding domain (RBD) of the spike protein on SARS-CoV-2 that binds to the angiotensin-converting enzyme 2 (ACE2) receptor expressed on host cells (3–5). While not all antibodies against the RBD are neutralizing (6, 7), these test values may reflect the proportion of antibodies that protect against SARS-CoV-2 (8, 9). Therefore, any correlation between commercial test results and protective function against SARS-CoV-2 is of epidemiological and clinical interest.

Several SARS-CoV-2 variants have been identified (10). The B.1.351 variant, originally identified in South Africa, is characterized by amino acid mutations such as K417N, E484K, and N501Y in the RBD of the spike protein (10). These mutations can alter neutralization by antibodies against earlier strains of COVID-19 as well as viral binding, because of structural changes in its sites contacting the ACE2 receptor (11). The B.1.1.7 variant that emerged in the United Kingdom also has the mutation N501Y (12), which has been shown to increase affinity for the ACE2 receptor (13). It has been suggested that N501Y and the other mutations in the B.1.1.7 variant are not related to reduced neutralization (14, 15); however, reduced neutralization has also been observed (16, 17). Thus, elucidating whether antibodies present in the sera from COVID-19 patients have neutralizing activity against emerging SARS-CoV-2 variants and whether the commercial antibody test reflects the neutralizing activity against them remains paramount.

The optimization of immune response tests may help the accurate evaluation of infection-induced antibodies as well as the efficacy of vaccination. Using sera from COVID-19 patients, individuals reporting episodes of close contact with COVID-19, and suspected healthy individuals, the performance of the CRNT to recognize individuals who have likely acquired immunity was evaluated. In addition, we investigated whether these neutralizing effects could be predicted by a commercial antibody test.

## RESULTS

### Clinical findings and antibody responses.

**FIG 1:**
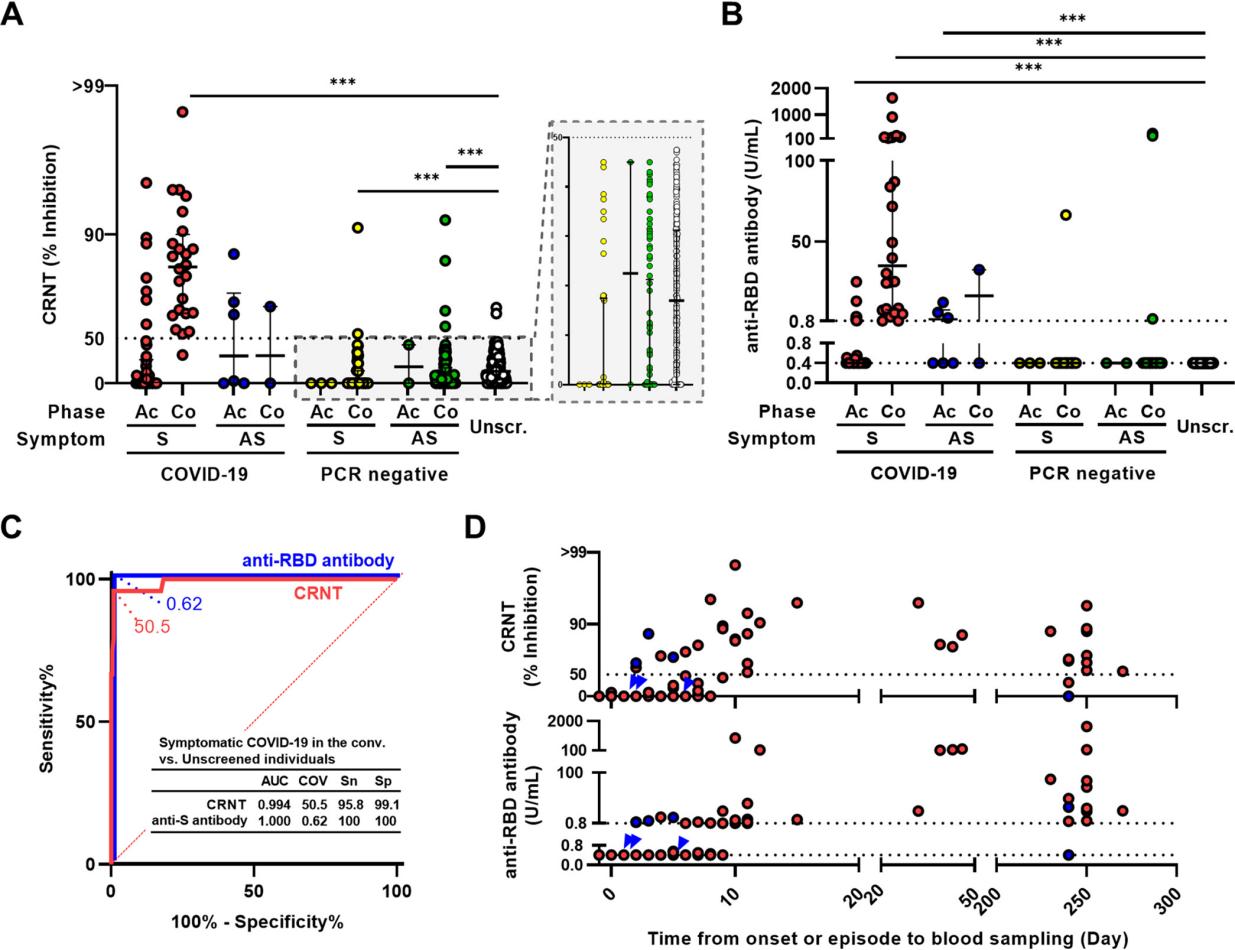
Neutralization and anti-RBD antibody levels. (A) Neutralization of pseudotyped viruses measured by CRNT (serum dilution, ×100). (B) Anti-RBD antibody levels measured by commercial test. (C) ROC curves to classify the symptomatic confirmed COVID-19 patients in the convalescent phase and the unscreened individuals. (D) Relationship between test results and time from symptom onset or close contact to blood sampling in COVID-19 patients. Symptomatic and asymptomatic individuals are presented in red and blue (blue arrowhead for overlapping cases), respectively. ***, *P* < 0.001 by unpaired Kruskal-Wallis test and Dunn’s multiple comparison using the unscreened group as control. Ac, acute phase; Co, convalescent phase; S, symptomatic; AS, asymptomatic; Unscr., unscreened; PCR negative, SARS-CoV-2 PCR negative; AUC, area under the curve; COV, cut-off value; Sn, sensitivity; Sp, specificity.

To investigate the relationship between clinical findings and seroconversion, 482 serum samples, excluding three samples with low volumes remaining, were evaluated. These samples were collected from confirmed COVID-19 patients (*n* = 74), SARS-CoV-2 PCR-negative individuals (*n* = 179), and unscreened individuals (*n* = 229) (Table 1). Because in our previous study, patients with moderate and severe COVID-19 showed >50% inhibition (IC_50_) in the CRNT after day 10 from disease onset (2), the period after day 10 from disease onset was defined as the convalescent phase. For SARS-CoV-2 PCR negative individuals, in order to consider the false-negatives for PCR in both symptomatic and asymptomatic groups, the period after day 10 from the episode of close contact was also defined as the convalescent phase. Neutralization activity against pseudotyped viruses and anti-RBD antibody levels were evaluated by the CRNT (Fig. 1A) and quantified by the commercial test (Fig. 1B). The serum dilution for CRNT was set to 100-fold because values from 100-fold dilutions had exceeded IC_50_ with a higher rate than those of 400-fold dilutions of the sera of convalescent patients with symptomatic COVID-19 that were expected to be positive (Fig. S1A). Similarly, the anti-RBD antibody test using undiluted sera routinely yielded values of >0.8 U/mL compared with the same sera when diluted (Fig. S1B). Because asymptomatic individuals can have a weak immune response to SARS-CoV-2 infection (18), the diagnostic performance of both tests was evaluated by comparing the results for patients confirmed to have symptomatic COVID-19 in the convalescent phase (*n* = 24) with unscreened individuals (Fig. 1C). Both tests clearly discriminated between these two groups (best COVs: CRNT, 50.5; anti-RBD antibody test, 0.62). Thus, in the following analysis, the IC_50_ for the CRNT and 0.8 U/mL for the anti-RBD antibody test (manufacturer’s COV) were used as COVs for predicting seroconversion.

**TABLE 1:** Demographic and clinical characteristics of study participants

Profile	Confirmed COVID-19, *n* = 74	SARS-CoV-2 PCR negative, *n* = 179	Unscreened, *n* = 229
Sex, male, *n* (%)	32 (43.2)	60 (33.5)	101 (44.1)
Age, yrs, *n* (%)			
≤19	4 (5.4)	1 (0.6)	0 (0.0)
20–29	14 (18.9)	42 (23.5)	51 (22.3)
30–39	11 (14.9)	51 (28.5)	71 (31.0)
40–49	6 (8.1)	39 (21.8)	53 (23.1)
50–59	10 (13.5)	25 (14.0)	35 (15.3)
60–69	9 (12.2)	11 (6.1)	19 (8.3)
≥70	20 (27.0)	10 (5.6)	0 (0.0)
Symptom, *n* (%)			
Symptomatic	66 (89.2)	56 (31.3)	NA^*a*^
Fever	50 (67.6)	22 (12.3)	
Cough	43 (58.1)	22 (12.3)	
Sputum	18 (24.3)	17 (9.5)	
Sore throat	15 (20.3)	19 (10.6)	
Nasal discharge	7 (9.5)	13 (7.3)	
Loss of taste	22 (29.7)	2 (1.1)	
Loss of smell	25 (33.8)	3 (1.7)	
Dyspnea	21 (28.4)	11 (6.1)	
Others	19 (25.7)	12 (6.7)	
Asymptomatic	8 (10.8)	123 (68.7)	NA
Phase of blood sampling, *n* (%)			
Acute (<9 days from onset or a close contact episode), *n* (%)	48 (64.9)	5 (2.8)	NA
Convalescent (≥10 days from onset or a close contact episode), *n* (%)	26 (35.1)	174 (97.2)	NA
Underlying diseases, *n* (%)			
Yes	14 (18.9)	10 (5.6)	NA
Malignant diseases	2 (2.7)	4 (2.2)	
Diabetes	8 (10.8)	7 (3.9)	
Immunosuppression	3 (4.1)	1 (0.6)	
Renal failure	5 (6.8)	2 (1.1)	
Liver failure	0 (0.0)	1 (0.6)	
Systemic lupus erythematosus	0 (0.0)	0 (0.0)	
No	60 (81.1)	169 (94.4)	NA
Medication, *n* (%)			
Yes	5 (6.8)	7 (3.9)	NA
Corticosteroids (excluding ointment)	3 (4.1)	6 (3.3)	
Immunosuppressants	1 (1.4)	1 (0.6)	
Anti-tumor drugs	0 (0.0)	0 (0.0)	
Anti-rheumatoid drugs	1 (1.4)	1 (0.6)	
Radiological therapy	0 (0.0)	1 (0.6)	
No	69 (93.2)	172 (96.1)	NA

a NA, not applicable.

For the CRNT (Fig. 1A and Table S1), symptomatic patients with confirmed COVID-19 were 16.7% (7/42) in the acute phase and 95.8% (23/24) in the convalescent phase (*P* < 0.01, Table S2). The CRNT values for the symptomatic patients with confirmed COVID-19 (median 83.5; IQR 64.1–90.0) were significantly higher than those of the unscreened individuals (% positivity: 0.9% [2/229], median 17.0; IQR 0.0–31.2) (*P* < 0.001). Conversely, symptomatic and asymptomatic SARS-CoV-2 PCR-negative individuals in the convalescent phase showed significantly lower CRNT values than those of the unscreened individuals (*P* < 0.001).

For anti-RBD antibody levels (Fig. 1B and Table S1), in the symptomatic confirmed COVID-19 patients, 14.3% (6/42) in the acute phase and 100.0% (24/24) in the convalescent phase tested positive (*P* < 0.01, Table S2). In contrast, 0.0% (0/229) of the unscreened individuals were positive. Compared with the unscreened group, symptomatic patients with confirmed COVID-19 in the acute phase, those in the convalescent phase, and asymptomatic patients with confirmed COVID-19 in the acute phase showed significant increases in serum anti-RBD antibody levels (median 0.40, IQR 0.40–0.40; median 0.40, IQR 0.40–0.46; median 35.0, IQR 7.63–137.0; and median 1.59, IQR 0.40–7.55, respectively; *P* < 0.001). There were no significant differences between the SARS-CoV-2 PCR-negative and unscreened groups.

Among the confirmed COVID-19 patients, seroconversion was observed 2 days after onset or the episode of close contact; moreover, patients in the convalescent phase were positive for both tests, excluding two patients sampled after 240 days (Fig. 1D). All patients with COVID-19 who were CRNT-positive in the acute phase were also positive for anti-RBD antibody, regardless of symptoms (Table S4). While the confirmed COVID-19 group included subpopulations with RNAemia, it was independent of seroconversion assessed by the neutralization or anti-RBD antibody tests during the acute phase (Fig. S2).

### Relationship between anti-RBD antibody and neutralization tests.

**FIG 2:**
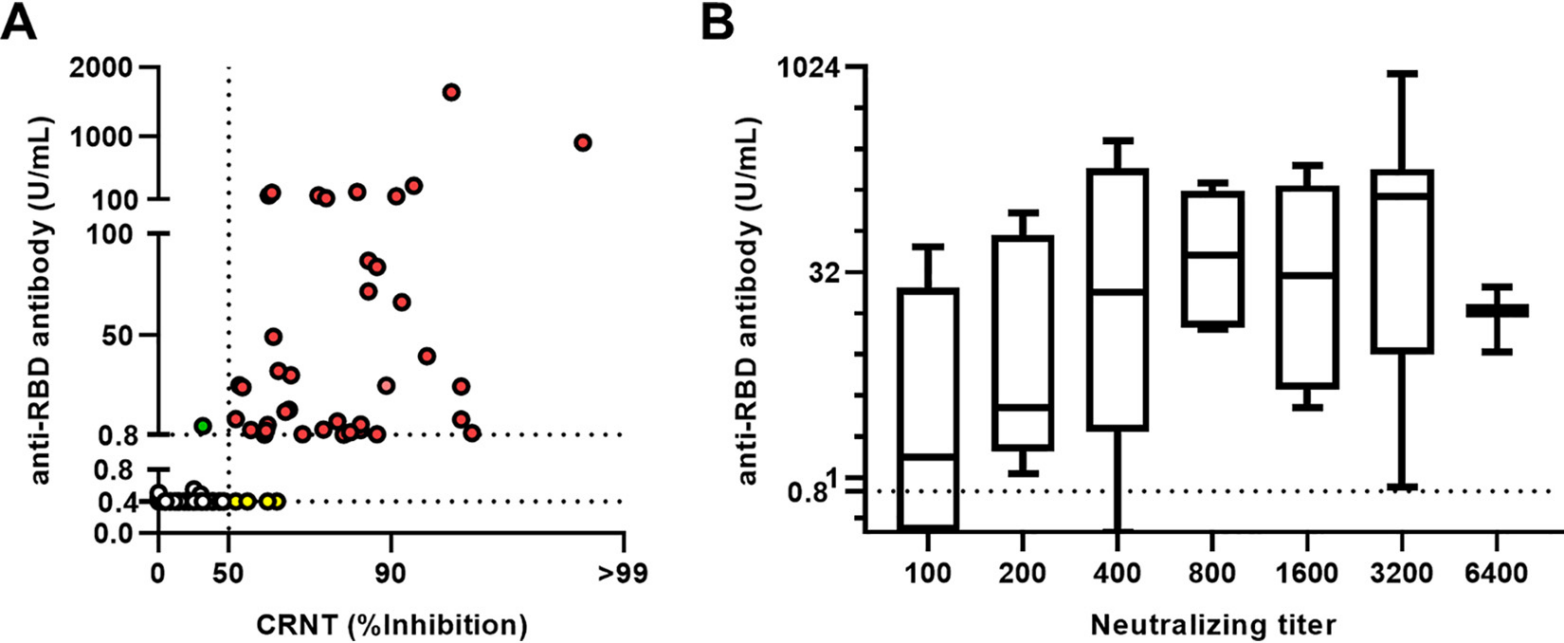
Relationship between CRNT and anti-RBD antibody test. (A) Comparison of neutralization levels and anti-RBD antibody results. Concordant samples are red (positive for both tests) or white (negative for both tests). Discordant samples are green (positive for anti-RBD antibody) or yellow (positive for CRNT). Dotted line for CRNT indicates 50% infectivity (IC_50_). (B) Anti-RBD antibody test as a function of neutralizing activity. Sera positive for CRNT (diluted 1:100) were serially diluted up to 1:6,400 and the dilution yielding >IC_50_ was defined as neutralizing titer. Boxes indicate median and interquartile. Error bars indicate minima and maxima.

To evaluate the functional significance of the more indirect anti-RBD antibody test, these values were compared with those obtained with the CRNT. Their concordance was 98.9% (477/482 double positive; *n* = 38; double negative: *n* = 439) (Fig. 2A). Of five discordant samples, four were slightly positive by CRNT (53.5–69.0) but negative by the anti-RBD antibody test (< 0.40 U/mL). For the other discordance, for which the anti-RBD antibody test yielded a slightly elevated 5.06 U/mL, CRNT yielded partial inhibition (CRNT 35.5), but was judged to be negative. Discordance was not related to underlying diseases or medications.

Next, to evaluate whether the anti-RBD antibody results indirectly correlated with neutralization tests, these values were compared with CRNT using diluted sera (Fig. 2B). The results were positively correlated (*R* = 0.31, 95% CI 0.22 to 0.38, *P* < 0.01); the sera with higher values tended to be positive for CRNT (>50% inhibition) despite being highly diluted.

### High-throughput CRNT (htCRNT).

To increase the number of simultaneous processing, the performance of htCRNT, which is a high-throughput neutralizing assay on a 384-well plate, was also evaluated using 100-fold-diluted sera. The results from htCRNT correlated with those of CRNT (*R* = 0.72, 95% CI 0.67 to 0.76, *P* < 0.01), with 98.8% concordance (476/482). Six discordant samples were negative for htCRNT but positive for CRNT (Fig. S3A and Table S2). Significant inhibition was observed in sera from patients with symptomatic confirmed COVID-19 in the convalescent phase (median 83.5; IQR 67.7–91.0) compared with unscreened individuals (median 0.0; IQR 0.0–11.0; *P* < 0.001; Fig. S3B). The cut-off value was set as the IC_50_ based on ROC analysis (Fig. S3C); then, the concordance was 99.4% (479/482 double positive, *n* = 36; double negative: *n* = 443; *R* = 0.37, 95% CI 0.30 to 0.45, *P* < 0.01; Fig. S3D). Three discordant samples were positive by anti-RBD antibody test but negative by htCRNT (htCRNT 28–41). Three patients in the convalescent phase were negative by htCRNT (Fig. S3E).

### Cross-reactivity with pseudotyped SARS-CoV-2 variants.

**FIG 3:**
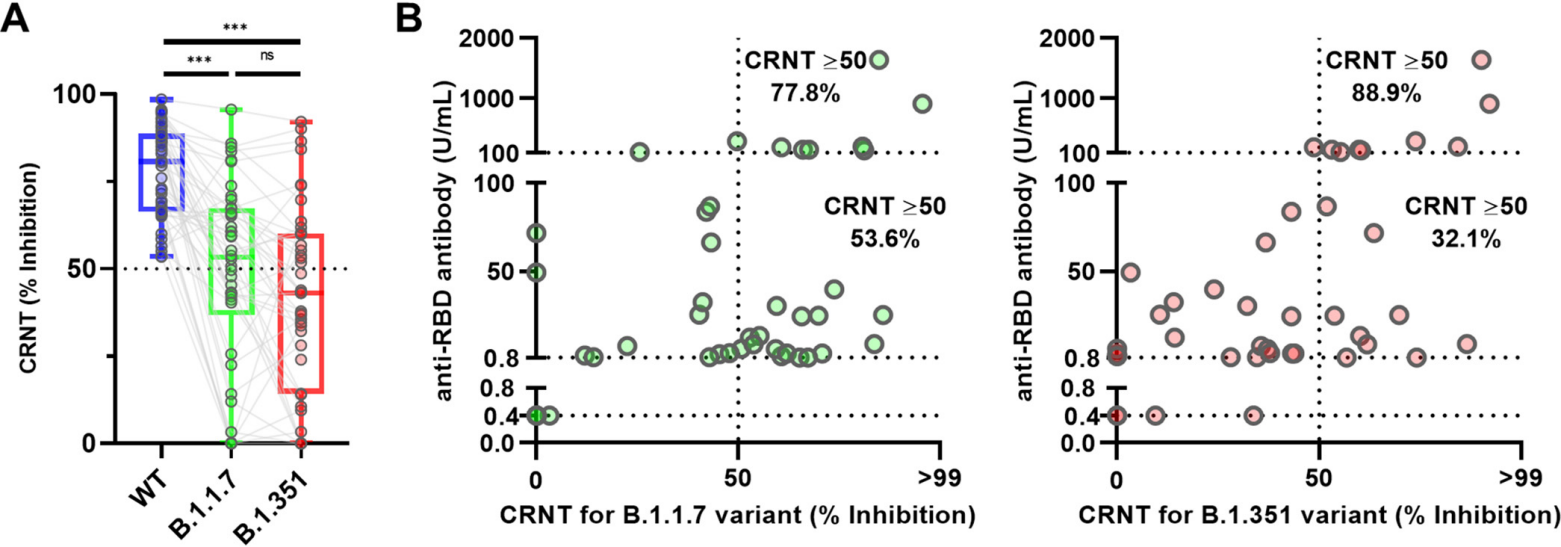
Neutralizing activities against SARS-CoV-2 variants in Wuhan-CRNT-positive sera. (A) Neutralizing sensitivity of SARS-CoV-2 pseudotyped variants. Neutralization by wild-type (WT) spike protein (Wuhan) CRNT-positive sera (serum dilution, ×100) was assessed against pseudotyped viruses displaying the mutant spike proteins (B.1.1.7 and B.1.351-derived variants). Box indicate median and interquartile. Error bars indicate minimum to maximum. ***, *P* < 0.001; ns, not significance. (B) Relationship between anti-RBD-antibody test results and neutralization of B.1.1.7 and B.1.351 variants (serum dilution 1:100).

Finally, to measure the neutralization of SARS-CoV-2 variants, CRNT-positive samples were assayed using pseudotyped B.1.1.7- and B.1.351-derived variants. Compared with the WT pseudotyped virus (Wuhan), the CRNT values for neutralization of the B.1.1.7- and B.1.351-derived variants were significantly decreased (WT median 80.8, IQR 66.4–88.8; B.1.1.7 median 53.4, IQR 36.7–67.4; B.1.351 median 43.1, IQR 14.2–60.2) (Fig. 3A). The CRNT results against both variants was positively correlated with those from the anti-RBD antibody test (B.1.1.7, *R* = 0.36, 95% CI 0.06 to 0.60, *p* < 0.05; B.1.351, *R* = 0.49, 95% CI 0.22 to 0.69, *P* < 0.01) (Fig. 3B). The percentages of serum samples above CRNT 50.0 were 77.8% (7/9) for B.1.1.7 and 88.9% (8/9) for B.1.351 for samples with ≥100 U/mL by the anti-RBD antibody test, while they were 53.6% (15/28) for B.1.1.7-derived variant and 32.1% (9/28) for B.1.351-derived variant among samples with 0.8–<100 U/mL by the anti-RBD antibody test (B.1.1.7, *P* = 0.26; B.1.351, *P* < 0.01; Fisher’s exact test) (Fig. 3B). Exceeding 100 U/mL in anti-RBD antibody test was associated with the ability to neutralize these variants (*P* < 0.01, chi-square test) (Table 2).

**TABLE 2:** Relationships between anti-RBD antibody tests and CRNT using the B.1.1.7- and B.1.351-derived variants

Anti-RBD antibody test	CRNT results against the B.1.1.7- and B.1.351-derived variants (*n*)			Chi-square test
	Negative for both	Positive for one variant	Positive for both	
≥100 U/mL	0	3	6	*p* < 0.01
<100 U/mL	12	14	12	

For the CRNT-positive samples, CRNT and anti-RBD data were compared with those of enzyme-linked immunosorbent assay (ELISA) for each spike variant antigen (Fig. S4A and B). The values from ELISA for the variants had similar ranges as those for WT. Some samples with relatively high ELISA values had values less than the CRNT IC_50_ for each variant; some with relatively low values exceeded the CRNT IC_50_.

## DISCUSSION

*In vitro* neutralization can be predictive of immune protection against SARS-CoV-2 infection (19); however, neutralization assays are not suitable for clinical laboratories. Therefore, it is important to determine whether the clinical anti-RBD antibody test accurately predicts protection, because not all antibodies against the RBD are neutralizing (6, 7). In the present study, results from the anti-RBD antibody test and CRNT positively correlated. This finding is consistent with previous reports (20–22), suggesting that the anti-RBD antibody test reflects protective immunity. In addition, CRNT and anti-RBD antibody testing successfully discriminated between symptomatic patients with COVID-19 patients in convalescence and suspected healthy individuals, suggesting that both tests are suitable for detecting SARS-CoV-2 infection. However, there were also seropositive individuals in the SARS-CoV-2 PCR negative and unscreened groups. They might have seroconverted after an underdiagnosed SARS-CoV-2 infection, or they may have antibodies that cross-react with the SARS-CoV-2 spike protein, but had been elicited by infection by other human coronaviruses (HCoVs) such as HCoV-OC43, -229E, -NL63, and -HKU1 (23, 24).

The strength and duration of long-term persistence of effective immunity after recovery from COVID-19 remains controversial. While early reports indicated sustained humoral immunity in sera from previously infected patients (9, 25), SARS-CoV-2 reinfection has been reported despite this immune response (26, 27). Although neutralizing antibodies may disappear after 6 months or longer after onset (4), in the present study most of the convalescent-phase sera from COVID-19 patients exhibited neutralization activity. Asymptomatic COVID-19 patients may have a weaker immune response than symptomatic patients or have reduced anti-RBD antibody and neutralizing antibody levels, as previously reported (18). Conversely, seropositivity in the hyperacute phase may represent cross-reacting antibodies because they are present earlier than they should be, given our general understanding of the kinetics of the immune response (28). However, clinical findings, including quantitative PCR suggested SARS-CoV-2 infection. They might have already been exposed to the virus without noticing the onset date or any close contacts with infected individuals. These findings support the existence of long-term immunity against SARS-CoV-2, particularly in symptomatic patients; however, continuous precautions, including infection control and prevention, and vaccination, are required for reliable immunity, as convalescent-phase sera can contain insufficient levels of neutralizing activity (25).

It is important that any immunity acquired against WT SARS-CoV-2 has sufficient cross-reactivity against variants. In the present study, reduced neutralization was observed for the B.1.1.7- and B.1.351-derived variants compared with the WT, consistent with other reports (16, 17, 29). For the B.1.1.7-derived variant, although there are also reports that variant neutralizing activity is similar to WT (14, 29, 30), our findings support the systematic report (29). In addition, in the analysis limited to CRNT-positives, it could be difficult to detect any reduction in neutralization against variants by the anti-RBD antibody test and ELISA, because the values for the reduced neutralization population clearly overlapped those for >IC_50_ population. Our findings also suggest the presence of neutralizing activity against variants when the anti-RBD antibody test provides a relatively high value. However, it should also be noted that those samples that were above the IC_50_ did not always exceed 100 U/mL in the anti-RBD antibody test.

The high-throughput microassay (htCRNT) showed a good correlation with the CRNT. Because it requires a smaller volume of serum and virus than the standard CRNT, a larger number of samples can be simultaneously assayed. Furthermore, because the neutralizing antibody test is the gold standard for assessing functional immune status against the virus, it can provide evidence for planning the resumption of social activity. There are many issues requiring the measurement of immune status, such as the antibody response of health care workers after vaccination, and the antibody retention ratio in the community. Therefore, a high-throughput option increases the scale screening. However, considering the slope of the correlation, the values of htCRNT may be slightly lower than those of CRNT. Although we could not determine whether the samples that did not match between the two tests were false-positives for CRNT or false-negatives for htCRNT, when htCRNT is used for screening, CRNT-positives yielding relatively low values might be missed.

There are several limitations to the present study. First, the serum samples were one-time collections. Therefore, the continuous antibody level trend and its relationship with disease severity could not be evaluated. Second, sera from individuals who had no evidence of infection, such as sera sampled before the COVID-19 pandemic, could not be used as controls.

In conclusion, both the CRNT and anti-RBD antibody tests efficiently detect immune responses convalescent COVID-19 patients. Because most facilities cannot evaluate neutralizing antibodies, the good correlation of the nonfunctional antibody test with the CRNT may help assess the levels of functional antibodies in patients with COVID-19 and vaccinated individuals.

## MATERIALS AND METHODS

### Specimen collection.

Serum samples were collected from COVID-19 patients, uninfected close contacts, and suspected healthy individuals at the Toyama University Hospital and Toyama City Hospital. Sera were frozen at −80°C until assayed. All samples were collected before SARS-CoV-2 variants had been detected locally.

COVID-19 diagnoses were confirmed by positive nasopharyngeal swab samples in the SARS-CoV-2 quantitative reverse transcriptase–PCR (RT-qPCR) test, hereafter referred to as confirmed COVID-19 patients. The uninfected close contacts, including health care workers, were those who were negative at least once with RT-qPCR are hereafter referred to as SARS-CoV-2 PCR-negative. Suspected healthy individuals (hereafter referred to as unscreened) were health care workers at the Toyama University Hospital who had not been tested by RT-qPCR, as they were not considered at risk and had neither presented with symptoms nor reported close contacts.

Basic clinical characteristics were obtained from medical records or questionnaires from all participants who had been tested by RT-qPCR. These included symptoms (fever, cough, sputum, sore throat, nasal discharge, loss of taste, loss of smell, dyspnea, and others), underlying diseases (malignancies, diabetes, immunosuppression, renal failure, liver failure, and systemic lupus erythematosus), and medications (corticosteroids excluding ointment, immunosuppressants, anti-tumor drugs, anti-rheumatoid drugs, and radiological therapy).

For suspected healthy (unscreened) individuals, information on symptoms, underlying diseases, and medications was not collected. Serum samples from these individuals were originally collected in July 2020 and August 2020 for the screening of subclinical SARS-CoV-2 infections among staff by the infection-control team because at least 3 months had passed since the first patient with COVID-19 had been hospitalized.

### Virological investigation.

SARS-CoV-2 RT-qPCR was performed at officially approved laboratories, including the University of Toyama, Toyama Institute of Health, and external private laboratories. The RT-qPCR varied by laboratory. Cases of RNAemia were screened (31), with the results used as the demographic background. When the remaining respiratory specimens were available, co-infecting microorganisms were screened using the FilmArray Respiratory Panel 2.1 (bioMérieux Japan, Tokyo, Japan), according to the manufacturer’s instructions.

### Generation of pseudotyped viruses.

Pseudotyped vesicular stomatitis virus (VSV) bearing SARS-CoV-2 spike (S) protein was generated as previously described (1). The expression plasmid for the truncated S protein of SARS-CoV-2, pCAG-SARS-CoV-2 S (Wuhan), was kindly provided by Dr. Shuetsu Fukushi, National Institute of Infectious Diseases, Japan. The expression plasmids for the truncated mutant S protein of SARS-CoV-2, pCAGG-pm3-SARS2-Shu-d19-B1.1.7 (UK-derived variant) and pCAGG-pm3-SARS2-Shu-d19-B1.351 (South Africa-derived variant), were constructed by PCR-based site-directed mutagenesis using the cDNA as a template, which had been produced by chemical synthesis with human codon optimization (Thermo Fisher Scientific, MA, USA). The S cDNA of SARS-CoV-2 was cloned into the pCAGGS-pm3 expression vector. Briefly, 293 T cells were transfected with the above expression vectors. After 24 h of incubation, transfected cells were infected with G-complemented (*G) VSVΔG/Luc (*G-VSVΔG/Luc) at a multiplicity of infection of 0.5. The virus was adsorbed, then extensively washed four times with Dulbecco’s modified Eagle’s medium (DMEM) supplemented with 10% fetal bovine serum (FBS). After a second 24 h of incubation, culture supernatants containing pseudotyped VSVs were centrifuged to remove cell debris and stored at −80°C for later use.

### Serology.

Serum neutralization against pseudotyped viruses was assayed in 96-well microplates (Thermo Fisher Scientific, MA, USA) using the CRNT, as previously described (1). In this study, we used VeroE6/TMPRSS2 cells (JCRB1819), which are highly susceptible to SARS-CoV-2 infection. They were purchased from the Japanese Collection of Research Bioresources (JCRB) Cell Bank (Osaka, Japan). Briefly, serum samples were mixed with DMEM (Nacalai Tesque, Inc., Kyoto, Japan) supplemented with 10% heat-inactivated FBS by serial dilution and incubated with pseudotyped SARS-CoV-2 for 1 h. This mixture was incubated with VeroE6/TMPRSS2 cells. Infectivity was quantified by measuring luciferase activity after 24 h of incubation at 37°C and expressed as the mean of duplicate measurements. For the high-throughput assay (htCRNT), the CRNT was modified to use 384-well microplates (Corning, NY, USA).

For the commercial assay, serum samples were tested at an external private laboratory, using the Elecsys Anti-SARS-CoV-2 S immunoassay (Roche Diagnostics GmbH, Basel, Switzerland) to quantify antibodies recognizing the SARS-CoV-2 RBD. The manufacturer’s cut-off value (COV) was 0.8 U/mL and the minimum value was expressed as <0.4 U/mL.

For ELISA, 30 ng of histidine-tagged recombinant SARS-CoV-2 S proteins of three genotypes: WT, B1.1.7 variant (N501Y), and B.1.351 variant (K417N, E484K, and N501Y) were immobilized in triplicate on Immulon 2 HB 96-well microtiter plates (Thermo Fisher Scientific, MA, USA). Plates were blocked with 1% bovine serum albumin at 37°C overnight, then incubated with 100 μl of 1:10 diluted serum or 1.0 μg/mL mouse anti-His_6_ antibody (BioDynamics Laboratory, Tokyo, Japan) at 37°C for 2 h. Peroxidase-conjugated AffiniPure goat anti-human or anti-mouse IgG (Jackson ImmunoResearch, PA, USA) were dispensed into each well and incubated at 37°C for 2 h. Color was developed with the SIGMAFAST OPD (Sigma-Aldrich, MO, USA) substrate for 5 min and the reaction was stopped by adding 3 N H_2_SO_4_. Absorbance was read at 490 nm. Each sample was tested in triplicate.

### Statistical analysis.

Statistical analysis was performed using the Kruskal-Wallis test with Dunn’s test for multiple comparisons among three groups or more. Correlations between test findings were determined using Pearson’s correlation coefficients. Positive conversion was analyzed by the Kaplan-Meier method, using the Gehan-Breslow-Wilcoxon test. Data were analyzed using GraphPad Prism version 8.4.3 (GraphPad Software, CA, USA). Fisher’s exact and chi-square tests were performed using QuickCalcs (GraphPad Software, CA, USA; https://www.graphpad.com/quickcalcs/). Statistical significance of differences between groups is presented in figure legends.

### Ethics approval.

This study was performed in accordance with the Declaration of Helsinki and was approved by the ethical review board of the University of Toyama (Approval No.: R2019167 and R2020097). Written informed consent was obtained from all participants.

### Data availability.

All data are provided in the manuscript and supplementary information.
