# Supplementary material for: Correlation of the Commercial Anti-SARS-CoV-2 Receptor Binding Domain Antibody Test with the Chemiluminescent Reduction Neutralizing Test and Possible Detection of Antibodies to Emerging Variants
Source: Microbiol Spectr. 2021 Dec 1;9(3):e00560-21. doi: 10.1128/Spectrum.00560-21 (PMC8635131; doi:10.1128/Spectrum.00560-21)

Supplemental Table 1. Basic statistics of SARS-COV-2 serological tests

Profile	Confirmed COVID-19				SARS-CoV-2 PCR negative				Unscreened
	symptomatic		asymptomatic		symptomatic		asymptomatic		
	acute	convalescent	acute	convalescent	Acute	convalescent	acute	convalescent	
N	42	24	6	2	3	53	2	121	229
CRNT (%)									
Minimum	0.0	35.5	0.0	0.0	0.0	0.0	0.0	0.0	0.0
25% Percentile	0.0	64.1	0.0	0.0	0.0	0.0	0.0	0.0	0.0
Median	0.0	83.5	34.5	34.7	0.0	0.0	22.5	0.0	17.0
75% Percentile	30.6	90.0	75.2	69.5	0.0	17.5	45.0	21.2	31.2
Maximum	95.5	98.5	86.5	69.5	0.0	91.0	45.0	92.0	19.0
htCRNT (%)									
Minimum	0.0	28.0	0.0	10.0	0.0	0.0	0.0	0.0	0.0
25% Percentile	0.0	67.2	0.0	10.0	0.0	0.0	0.0	0.0	0.0
Median	0.0	83.5	45.5	25.5	0.0	0.0	2.0	0.0	0.0
75% Percentile	15.0	91.0	86.0	31.0	0.0	0.0	4.0	0.0	11.0
Maximum	98.0	98.0	89.0	31.0	0.0	83.0	4.0	91.0	50.0
Anti-RBD antibody (U/mL)									
Minimum	0.40	0.84	0.40	0.40	0.40	0.40	0.40	0.40	0.40
25% Percentile	0.40	7.63	0.40	0.40	0.40	0.40	0.40	0.40	0.40
Median	0.40	35.0	1.59	16.4	0.40	0.40	0.40	0.40	0.40
75% Percentile	0.46	137.0	7.55	32.4	0.40	0.40	0.40	0.40	0.40
Maximum	25.1	1640	12.3	32.4	0.40	66.4	0.40	294.0	0.40

CRNT, chemiluminescent reduction neutralizing test; htCRNT, high-throughput CRNT, RBD, receptor-binding domain

Supplemental Table 2. Samples with discordant serological test results

Profile	CRNT		htCRNT		Anti-RBD antibody	
	Values (% Inhibition)	Results	Values (% Inhibition)	Results	Values (U/mL)	Results
Confirmed COVID-19						
symptomatic, convalescent	35.5	Negative	28.0	Negative	5.06	Positive
asymptomatic, convalescent	69.5	Positive	41.0	Negative	32.4	Positive
SARS-CoV-2 PCR negative						
symptomatic, convalescent	53.5	Positive	0.0	Negative	0.40	Negative
asymptomatic, convalescent	85.0	Positive	37.0	Negative	2.05	Positive
asymptomatic, convalescent	58.5	Positive	22.0	Negative	0.40	Negative
Unscreened						
	69.0	Positive	22.0	Negative	0.40	Negative
	66.0	Positive	37.0	Negative	0.40	Negative

CRNT, chemiluminescent reduction neutralizing test; htCRNT, high-throughput CRNT; RBD, receptor-binding domain

Supplemental Table 3. Testing in acute and convalescent phases

Test	Result	Phase		Fisher's exact test
		Acute (n)	Convalescent (n)	
CRNT	Positive	13	21	$p < 0.01$
	Negative	38	2	
htCRNT	Positive	13	20	$p < 0.01$
	Negative	38	3	
Anti-RBD antibody test	Positive	13	22	$p < 0.01$
	Negative	38	1	

CRNT; chemiluminescent reduction neutralizing test, htCRNT; high-throughput CRNT, RBD; receptor binding domain

Supplemental Table 4. Test results and clinical symptoms of confirmed COVID-19 patients with positive serological tests in the acute phase

Days after onset or the episode of close contact	Age range (years)	CRNT		htCRNT		Anti-RBD antibody test		Symptoms
		% Inhibition	Results	% Inhibition	Results	U/mL	Results	
2	40-49	60.0	Positive	81.0	Positive	3.26	Positive	Fever
4	≥70	72.5	Positive	73.0	Positive	13.1	Positive	Fever, dyspnea
6	≥70	76.0	Positive	63.0	Positive	1.07	Positive	Fever, cough
7	60-69	80.5	Positive	77.0	Positive	3.40	Positive	Fever
8	60-69	95.5	Positive	91.0	Positive	1.72	Positive	Fever, cough, sputum, loss of taste, loss of smell
9	50-59	89.5	Positive	98.0	Positive	25.1	Positive	Fever, cough
9	≥70	88.5	Positive	78.0	Positive	1.09	Positive	Fever
2	≤19	65.5	Positive	79.0	Positive	2.77	Positive	No symptom*
3	≥70	86.5	Positive	89.0	Positive	5.97	Positive	No symptom*
5	≤19	71.5	Positive	85.0	Positive	12.3	Positive	No symptom*

* No symptoms but consecutive positives in follow-up SARS-CoV-2 PCR tests. Discharged after confirming a PCR-negative result.

CRNT; chemiluminescent reduction neutralizing test, htCRNT; high-throughput CRNT, RBD; receptor binding domain

Supplemental Figure 1

(A) CRNT using 100-fold and 400-fold dilutions of sera from patients convalescing following symptomatic COVID-19. **(B)** Anti-RBD antibody test using 100-fold and 400-fold dilutions of sera from patients convalescing following symptomatic COVID-19. Bars indicate medians.

Supplemental Fig. 1

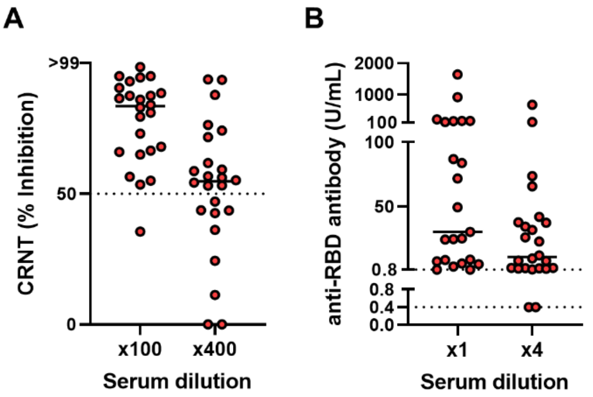

Supplemental Figure 2

Relationship between seroconversion and RNAemia in confirmed COVID-19 patients in the acute phase. The Kaplan-Meier analysis was performed only for patients who had RNAemia. ns, not significant.

Supplemental Fig. 2

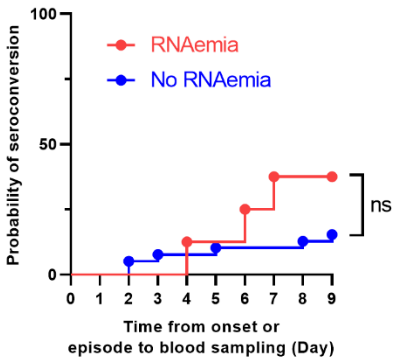

Supplemental Figure 3

(A) Correlation between htCRNT and CRNT. Neutralization was assessed using serum samples diluted 1:100. Correlation was expressed using Pearson's correlation coefficients. **(B)** Neutralization of pseudotyped viruses measured using htCRNT. **(C)** ROC curve of htCRNT for classification of symptomatic confirmed COVID-19 patients in the convalescent phase and unscreened individuals. **(D)** Comparison of htCRNT neutralization levels and anti-RBD antibody results. Concordant samples are red (positive for both tests) or white (negative for both tests). Discordant samples are green (positive for anti-RBD antibodies). The dotted line for htCRNT indicates 50% inhibition (IC_{50}). **(E)** Relationship between htCRNT results and time from symptom onset or a close-contact episode to blood sampling in patients with COVID-19. Symptomatic and asymptomatic individuals are presented in red and blue (blue arrowhead for overlapped), respectively. Error bars indicate medians (middle lines) and interquartile range. ***$p < 0.001$ by unpaired Kruskal-Wallis test and Dunn's multiple comparison (unscreened group as a control). Ac, acute phase; Co, convalescent phase; S, symptomatic; AS, asymptomatic; Unscr., unscreened; PCR negative, SARS-CoV-2 PCR-negative; AUC, area under the curve; COV, cut-off value; Sn, sensitivity; Sp, specificity.

Supplemental Fig. 3

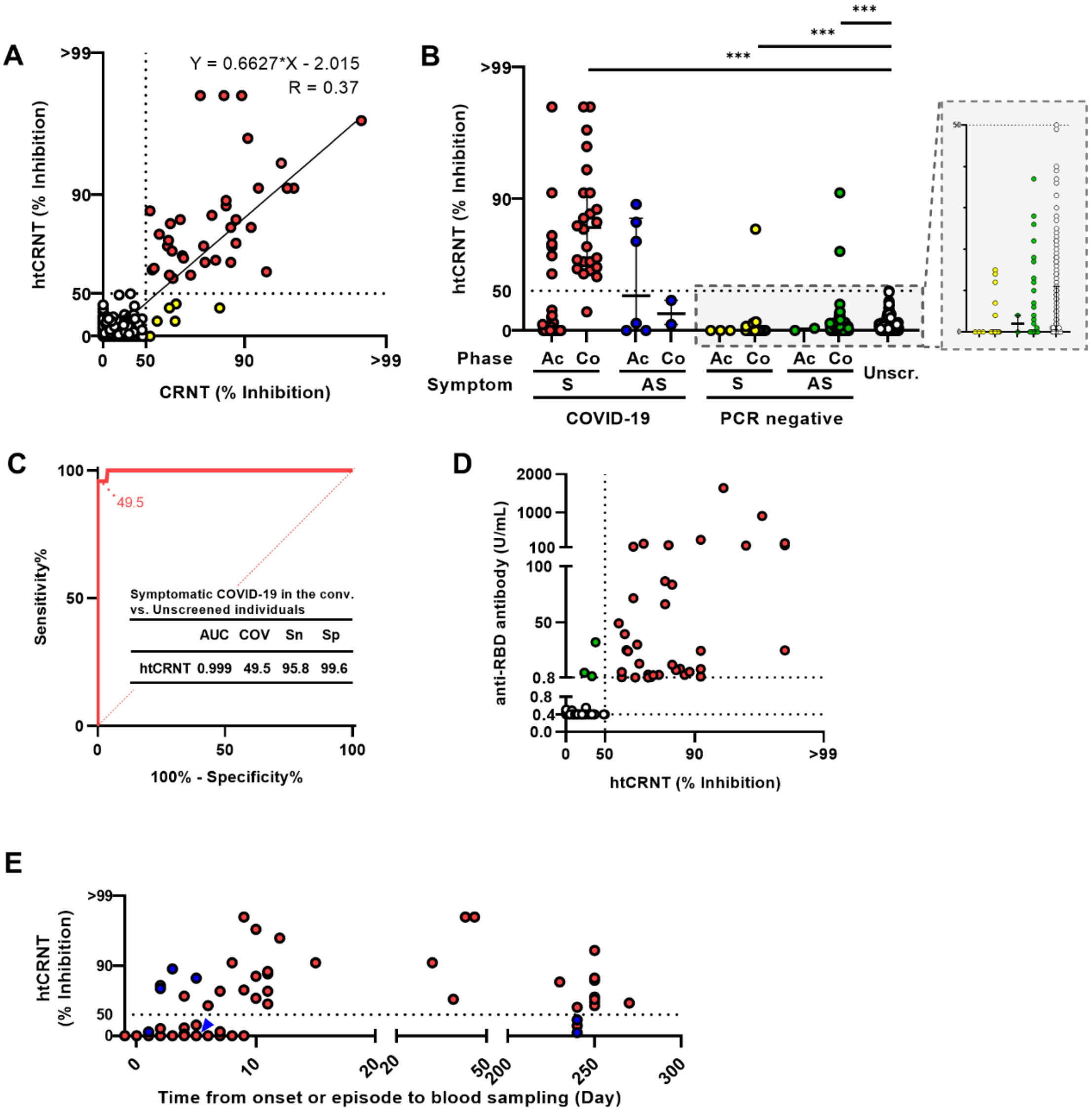

47

Supplemental Figure 4

Relationships between **(A)** ELISA and **(B)** CRNT values from sera that neutralize WT pseudotyped virus. The results from neutralization of WT (left), B.1.1.7 variant (middle), and B1.351 variant (right) were plotted for each. Dotted lines represent linear regression. Correlations were measured using Pearson's correlation coefficients.

Supplemental Fig. 4

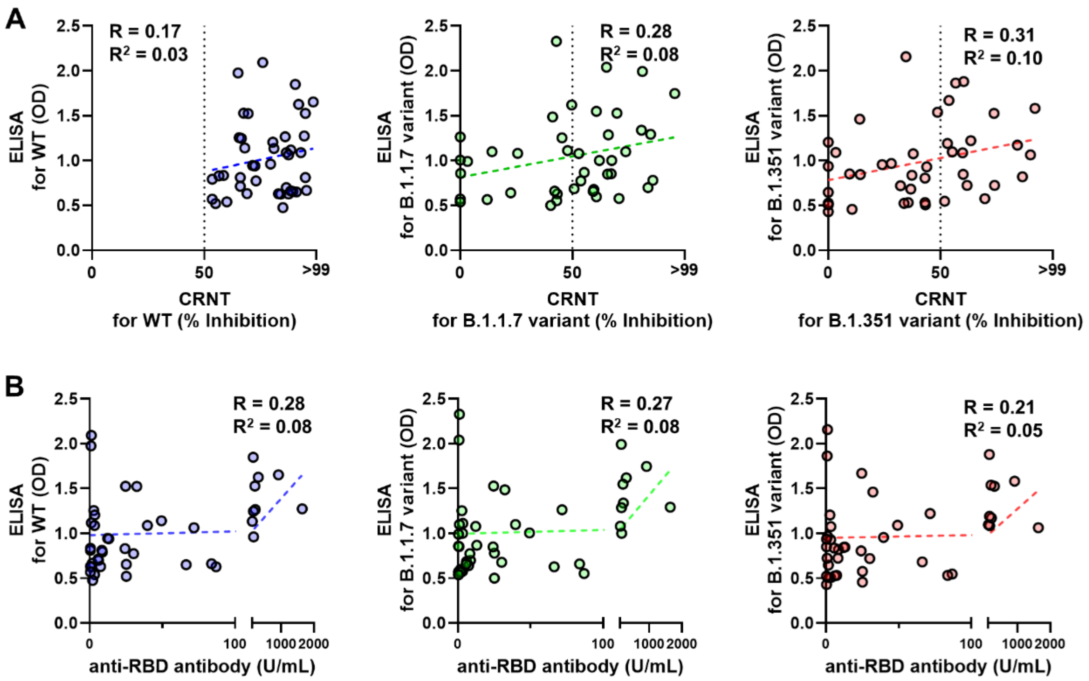

Supplement: SUPPLEMENTAL FILE 1 — Supplemental material. Download SPECTRUM00560-21_Supp_1_seq6.pdf, PDF file, 0.8 MB [file spectrum00560-21_supp_1_seq6.pdf]
